# Childhood Obesity: A Framework for Policy Approaches and Ethical Considerations

**Published:** 2011-08-15

**Authors:** Rogan Kersh, Donna F. Stroup, Wendell C. Taylor

**Affiliations:** New York University Wagner School, New York, New York; Data for Solutions, Inc; The University of Texas Health Science Center at Houston, School of Public Health, Houston, Texas

## Abstract

Although obesity rates among US children have increased during the past 3 decades, effective public policies have been limited, and the quest for workable solutions raises ethical questions. To address these concerns, in 2010, the Robert Wood Johnson Foundation convened an expert panel to consider approaches to the ethics problems related to interventions for childhood obesity. On the basis of recommendations from the expert panel, we propose frameworks for policy approaches and ethical aspects of interventions and evaluation. We present these frameworks in the context of other papers in this collection and make recommendations for public health practice.

## Introduction

Childhood obesity in the United States presents major health challenges, but neither the medical industry, public health advocates, nor policy makers have identified effective ways of reversing increasing rates of obesity among youth. Policy debates often focus on low energy expenditure attributable to increasingly inactive lifestyles. However, efforts to increase physical activity among youth have limited benefits without simultaneous attention to decreasing caloric consumption. A study among middle-school children reported that risk of obesity increased by 60% for every additional sugar-sweetened beverage consumed per day, regardless of levels of exercise (1).

Although obesity is linked to unhealthy diet and insufficient physical activity,  prevention efforts and responsibility for the problem remain controversial. Whose job is it to ensure that children have a healthy life: parents and caregivers, schools, communities, the state? Children may be particularly vulnerable to harm because of their limited ability to make choices, dependence on adults for food and other goods, and susceptibility to marketing.

The quest for solutions raises many ethical questions explored in this collection. Do interventions involving children raise concerns different from those for adults? Does public policy attention to childhood obesity exacerbate body-weight concerns that can fuel stigma and potentially cause bulimia and anorexia? In situations where multiple, simultaneous interventions on different levels are needed, how might testing a single intervention communicate misleading results about the efficacy of achieving sustainable reform?

In this commentary, we summarize recommendations of the expert panel. First, we present a policy framework for interventions for childhood obesity. Second, we develop a framework for addressing ethical issues. Third, we review 3 policy approaches to support this framework. Finally, we discuss the application of these frameworks for existing and planned interventions.

## Competing Policy Solutions

One perspective in US political discussions about childhood obesity emphasizes personal responsibility, holding that food consumption is an individual matter and that parents, and eventually adolescents themselves, are best situated to make consumption decisions (2,3). This view informs policy actions that emphasize improved access to volitional physical activity and healthy diets (4).

As concern over childhood obesity has increased, a competing policy framework has gained support. In an obesogenic environment, children may find their food choices influenced by availability, price, and marketing of high-fat, low-nutrient processed foods. Messages targeting youth start from an early age wherever they congregate, including at school (5). In this environmental view of childhood obesity, public officials have a responsibility to intervene through policies such as the following:

- Controlling the conditions of sale (eg, limiting what schools can offer).
- Restricting advertising of high-fat, low-nutrient foods that targets young children or using other alternatives to increase awareness of what they are eating (eg, requiring calorie labels on menus).
- Subsidizing healthier alternatives (eg, fruits and vegetables) that have much higher per-calorie costs than do most other foods, many of which are or include ingredients (eg, corn syrup and sugar) that are subsidized under US farm policies.
- Restricting or banning certain ingredients (eg, trans fats).

Policy initiatives to control availability of competitive foods have been introduced at all levels of government. One congressional bill expands the list of foods of minimal nutritional value forbidden for sale in school cafeterias and on campus (6). An example at the state level is Connecticut's Healthy Food Certification program, which provides monetary incentives to school districts that choose to implement state nutrition standards for all foods sold to students outside reimbursable school meals (7). The effort to assess and advance policy changes as discussed in this collection raises legitimate ethical concerns, to which we now turn.

## A Framework for Ethical Issues

**Box. Characteristics of State Interventions for Childhood Obesity Under the Stewardship Model**

Public health programs should

1. Attempt to reduce risks for obesity that populations might impose on each other.
2. Reduce causes of obesity through legislation or regulation that creates environmental conditions that sustain good health (eg, access to healthy foods and opportunities to be physically active).
3. Emphasize attention to the health of children and other vulnerable populations (eg, those with disabilities).
4. Promote health not only by providing information but also with programs that help populations maintain exercise and healthy diets.
5. Make leading a healthy life easy.
6. Ensure that populations have access to services.
7. Strive for justice in health.

Public health programs should not

1. Coerce populations into leading healthy lives.
2. Develop and introduce interventions without the consent or participation of those affected.
3. Implement interventions that are intrusive or conflict with personal or community values.

**Adapted from:** Nuffield Council on Bioethics (9).

One ethical concern raised by these policy interventions is the association between individual autonomy and state authority. The libertarian perspective limits the authority of the state to ensure individual freedom, whereas utilitarian and social-contract approaches allow individual interests to be secondary to increases in overall welfare. One theory for approaching this ethical concern is provided by John Stuart Mill's harm principle: state intervention is justified when a person's actions affect others (8). This principle recognizes the responsibility of the state to protect vulnerable populations from harming their own (or others') health. This harm principle can be applied to interventions for childhood obesity through a stewardship model (9), which argues that the state is a steward to people and communities (Box). A reasonable application of this stewardship role is the constitutional principle of police and public health authority explored by Harris and Graff in this collection (5).

The articles in this collection explore ethical questions about the role of the state or other societal structures in stewardship. For example, as the state attempts to protect school children by measuring and reporting body mass index (BMI), how can concerns about privacy and stigmatization be addressed (10)? Other questions concern the extent to which parents and other community members are responsible for providing children a safe environment and whether childhood obesity can be considered a child protection problem (similar to child abuse) needing societal intervention (11). Governments must prevent their actions from affecting certain communities disproportionately; for example, do taxes on sodas unjustly punish persons of lower socioeconomic levels (12)? Are state-provided interventions accessible to children with special health care needs (13)? How can we address the stigma associated with the use of the term "obese" (14)? Although food industry officials argue that marketing cannot force consumers to do anything, marketing continues to have a substantial impact. What should be the role of media literacy and restrictions on use of cartoon characters, celebrities, or health claims (5)? What can we learn from the restrictions on tobacco marketing (12)?

## Examples of Application of the Framework

We present 3 examples of policy interventions for childhood obesity to illustrate the application of these frameworks in light of the ethical issues explored in this collection.

### Menu calorie labeling

In 2008, no place in the United States required restaurants to post calorie labels. Two years later, dozens of jurisdictions and the United States itself have enacted menu-labeling laws (5). However, recent studies report that calorie information may not be a determining consideration in food choices; accessibility, taste, habit, perception, peer influence, and parental modeling also influence children's food choices (11). Thus, menu calorie labeling alone may not be effective, and communities considering this policy intervention would be well advised to consider the role of personal autonomy in implementing such interventions.

### Soft drink tax

Forty states and many cities levy taxes on low-nutrition foods (12). As for taxes to decrease cigarette consumption, controlled experiments have shown that manipulations of price can yield changes in consumption (15). The number of jurisdictions with soda taxes has declined in recent years concurrent with lobbying efforts by the beverage industry, but taxes have reduced consumption and increased revenue for other health-related programs (16). Just as for menu calorie labeling, the health benefits of a soda tax as a stand-alone intervention are less clear (17).

### Interventions in schools

The United States has built a public education system on the principle that no child should be denied the right to an education on the basis of socioeconomics or other challenges, yet when a child becomes obese, that child struggles to achieve academic success because of stigma, depression or anxiety, or absenteeism (18). Resources could be a factor in a school's reliance on unregulated foods to generate revenue (19). The ethical responsibility of schools to limit soft drink sales and provide healthy meals and opportunities for physical activity and to combat the other adverse consequences of childhood obesity affecting education (20) must also be considered.

The National School Lunch Program now serves more than 30 million students (approximately 60% of attendees) daily. Although students in this program consume more milk, fruits, and vegetables and have lower intakes of sweetened beverages and candy than other students, they also consume more sodium, fat and saturated fat, and calories (21). Moreover, US school districts often contract with private beverage and food companies to sell less nutritious "competitive foods" in cafeterias and vending machines. Thus, again, a stand-alone intervention may be ineffective, and the policy interventions planned for school settings must consider these competing forces.

The stewardship role of the state gives special attention to disadvantaged populations. Approximately 13% of children in the United States have a disability or chronic condition, and 6.4 million children with disabilities are enrolled in public education. Children with functional limitations and learning disabilities are more than twice as likely to be obese as other children, and children from families with low socioeconomic status are at higher risk for obesity (22). Parents, schools, health care settings, and communities all have a role in ensuring that the risk for obesity among children with special needs is no greater than for other children (13).

Ethical review of research protocols typically emphasizes informed consent and confidentiality, the standard in most research regarding health-policy interventions for human behavior. In their role as policy makers, school administrators may implement activities affecting children. Although schools are not a research setting, concern may be raised about the extent to which families are truly informed about the activities, a matter presumably heightened by any layer of removal (eg, child to parent or administrator) from the actual participant. The question of reporting BMI among school children illustrates this problem (10). School interventions that actively involve families are more likely to be effective (11) than stand-alone interventions in schools.

## Discussion

Media reports may exacerbate eating disorders and other unhealthy weight-loss practices. Reformers eager to spread a healthy message (eg, about the dangers of smoking, drinking, or drug use) have traditionally demonized both the purveyors of undesirable substances and those who practice the risky behavior, often targeting members of comparatively dispossessed communities (23). Unlike tobacco or drugs, food is necessary for life, so the attention to reducing stigma is a necessary component of any intervention (14).

This collection of papers supports the claim that the nature of evaluation research — testing a single intervention, often during the formative stage of implementation — may mislead policy makers and the public about the efficacy of achieving sustainable reform. If one focuses on a single isolated intervention and holds other factors constant (as if that were possible), the policy change may appear to be ineffective. When evaluations of individual policies (eg, menu labels, soft drink taxes, and removal of competitive foods in schools) fall short of anticipated benefits, does this imply that we are promoting the wrong policies or that no single intervention is likely to be successful in reversing the rates of childhood obesity? Or is our evaluation framework insufficient for this situation? A single type of medical treatment often fails to address a health problem, and multiple, simultaneous interventions are often preferable. A similar approach may be the most promising means of systematically addressing childhood obesity.

The advancement and impact of policy evaluations of simultaneous interventions face 2 challenges. First, scholars should find ways to evaluate broad interventions in scientifically sound ways and must attend  to collective concerns more rigorously. Evidence-based approaches (those informed by the best available scientific evidence and reflecting community preferences and feasibility) are more likely to be effective at addressing causes of childhood obesity, interventions, and policies that may work to confront those causes, in a manner acceptable to the community affected (24). Second, the separation of powers in the US legislative system, with its multiple veto points, combined with dedicated interest-group resistance to any attempts to regulate food or beverage policies, makes approval of passing even a single program difficult, much less a multifaceted, coordinated national approach to childhood obesity.

Given the urgency of the childhood obesity problem and the difficulty of personal-responsibility approaches (25), the public policy arena is the most promising response. Yet, in the United States, the time-honored policy-making practices of incrementalism are proving inadequate for the present crisis (26). For public policy to enable a response, barriers to simultaneous interventions, a new view of the role of the state, and attention to the ethical issues raised in this collection of articles will be needed.
